# Assessing the Effect of Twisting and Twisting Fatigue on ZnO:Al Thin Film Performance on PEN and PET Substrates

**DOI:** 10.3390/mi15070853

**Published:** 2024-06-29

**Authors:** Dilveen W. Mohammed, Rayan M. Ameen, Rob Waddingham, Andrew J. Flewitt, James Bowen, Stephen N. Kukureka

**Affiliations:** 1 School of Metallurgy and Materials, University of Birmingham, Edgbaston, Birmingham B15 2TT, UK; dilveen@uod.ac (D.W.M.); rayan@uod.ac (R.M.A.); 2 Department of Physics, University of Duhok, Zakho Way, Duhok P.O Box 78, Iraq; 3 Electrical Engineering Division, Department of Engineering, University of Cambridge, J J Thomson Avenue, Cambridge CB3 0FA, UK; 4 Department of Engineering and Innovation, Open University, Walton Hall, Milton Keynes MK7 6AA, UK

**Keywords:** polymer substrates, AZO, PET, PEN, twisting, flexible optoelectronic devices

## Abstract

This study examines the electromechanical characteristics of aluminium-doped zinc oxide (AZO) films. The films were produced using the RF magnetron sputtering process with a consistent thickness of 150 nm on various polymer substrates. The study focuses on assessing the electro-mechanical failure processes of coated segments using flexible substrates, namely polyethylene naphthalate (PEN) and polyethylene terephthalate (PET), with a specific emphasis on typical cracking and delamination occurrences. This examination involves conducting twisting deformation together with using standardised electrical resistance measurements and optical microscope monitoring instruments. It was found that the crack initiation angle is mostly dependent on the mechanical mismatch between the coating and substrate. Higher critical twisting angle values are observed for the AZO/PEN film during twisting testing. Relative to the perpendicular plane of the untwisted sample, it was found that cracks initiated at a twist angle equal to 42° ± 2.1° and 38° ± 1.7° for AZO/PEN and AZO/PET, respectively, and propagated along the sample length. SEM images indicate that the twisting motion results in deformation in the thin film material, leading to the presence of both types of stress in the film structure. These discoveries emphasise the significance of studying the mechanical properties of thin films under different stress conditions, as it can impact their performance and reliability in real-world applications. The electromechanical stability of AZO was found to be similar on both substrates during fatigue testing. Studying the electromechanical properties of various material combinations is important for selecting polymer substrates and predicting the durability of flexible electronic devices made from polyester.

## 1. Introduction

Flexible electronics currently attract substantial attention because they enable appealing new design options across large distances and areas. These devices demonstrate good structural integrity, low weight, and the possibility of affordable manufacturing using roll-to-roll methods [1]. Strong, light, and portable flexible electronic devices built on polymer substrates instead of brittle glass ones are achievable, and a great deal of design flexibility is available for flexible electronics fabricated on polymer substrates, which are sufficiently robust that they can be coiled up when not in use [2]. These features allow for the flexible design of a new generation of electronics that are very desirable for a variety of electronic products, including computers, wearables, toys, televisions, and smartphones [3]. Biaxially oriented polyethylene terephthalate (PET) and polyethylene naphthalate (PEN) films are now the most popular options for flexible electronic applications [4]. Thus, due to their lower material cost, lower deposition temperature, higher stability in environments containing activated hydrogen, higher optical transmission, and lower electrical resistivity when compared to those of indium tin oxide (ITO) and tin oxide (SnO_2_) films, transparent conducting ZnO films have drawn more attention [5]. These benefits are crucial for real-world uses including transparent electrodes, flexible flat panels that resemble paper, solar cells, and window material for displays. Numerous researchers have studied ZnO, a material that has been doped with aluminium to improve its electrical properties [3,6]. Methods, including pulsed laser deposition, electron-beam evaporation, direct-current (DC) magnetron sputtering, radio-frequency (RF) magnetron sputtering, and the sol-gel process, can be used to deposit aluminium-doped zinc oxide (AZO) films [7,8].

The low mechanical reliability of transparent conductive oxide (TCO) films, such as zinc tin oxide (ZTO) on polymer substrates, is one of the functional issues that poses a serious problem for flexible electronics operation and manufacturing processes. The brittle nature of TCO as an inorganic thin film makes it prone to cracking and/or buckling delamination when subjected to extensive mechanical strain, severely restricting the devices’ flexibility [7,9]. Consequently, these brittle films are the weakest components of flexible devices and the most likely sources of mechanical failure. Additionally, when the majority of this inorganic thin film is put onto a flexible substrate, substantial disparities in their coefficient of thermal expansion (CTE) and lattice mismatch result in significant residual stresses (due to CTE) and a faulty interface (due to mismatch) [10]. Hence, a good understanding of the individual mechanical properties of thin films and interface properties is the key for fabricating mechanically robust devices.

Tensile and bending tests have received the majority of attention in studies on the dependability of flexible electrodes. A brittle film subjected to tensile stressing conditions will first fracture at extremely low strains (<1%), and it will then continue to produce cracks with further strain until the saturation crack spacing is attained. Once the saturation spacing is achieved, the film may delaminate by buckling because fractures cannot grow between already-existing crack pieces [11].

The electromechanical characteristics of PET covered with ITO were examined by Cairns et al. [12] using a miniature tensile tester in conjunction with in situ optical microscopy. Between 2.0% and 2.5% strain, the initiation of cracking was observed; this results in an abrupt rise in the normalized electrical resistance.

Ni et al. [13] also examined the fracture characteristics of PET substrates coated with AZO under circumstances of simple-support bending. It was discovered that channel fractures initiate the damage to the coating under tensile strain, while the film specimen may delaminate from the polymer substrate and buckle prior to the commencement of a crack when subjected to compression.

In addition to tensile and bending deformations, the twisting deformations experienced by TCO films on interconnects can result in mechanical failures and contribute to an increase in electrical resistance. However, little attention has been directed to examine how TCO-coated polymer substrates behave mechanically under cyclic and monotonic twisting deformations. This is significant, as dynamic loading over numerous cycles can induce structural failure at stress levels lower than those required for failure under static loading conditions.

Lim et al. [10] investigated the electro-mechanical failure mechanisms of ZTO/Ag/ ZTO-coated PET utilizing a custom-built twisting apparatus in conjunction with optical microscopy and electrical resistance monitoring. The critical onset twisting angle for crack formation was identified as 38°. The authors noted that a conducting channel was aided by the overlapping films after the fracture occurred, which delayed the change in electrical resistance.

Mohammed et al. [14] observed that cracks began to form in the ITO/Ag/ITO/PET system at an angle of around 39°. SEM analysis revealed evidence of cracking and buckling delamination failures, suggesting the presence of both tensile and compressive stresses within the films. Additionally, they noted a notable increase in the percentage change in electrical resistance during cyclic fatigue testing at 100 °C compared to room temperature (RT) and 50 °C.

As described in the study conducted by Jung et al. [15], the twisting test was used to evaluate the mechanical durability of a flexible ITO/poly(3,4-ethylenedioxythiophene)–poly(styrenesulfonate) (PEDOT:PSS) film on a PET substrate. At a twisting angle of 46°, fractures in the PEDOT:PSS electrode were identified, although the normalized electrical resistance remained small until the twisting angle approached 50°.

The objective of this investigation is to examine the electro-mechanical flexibility of an AZO thin film coated onto PET and PEN substrates, particularly under conditions of twisting and twisting fatigue. It is anticipated that the results of this research will enhance our comprehension of the robustness and consistency of flexible electronic components under twisting deformation. This, in turn, will promote the commercialization and mass manufacture of such components.

## 2. Experimental Details

### 2.1. Substrates

Two semi-crystalline polyesters were used as substrates, namely 0.125 mm thick polyethylene terephthalate (PET Melinex ST 504) and 0.125 mm thick polyethylene naphthalate (PEN Teonex Q65FA). Both were provided in the form of A4 sheets as samples (DuPont Teijin Films, Dumfries, UK). Sheets were sectioned into samples of 30 mm in length, 18 mm in gauge, and 4 mm in width by using a Moore Hydraulic Press.

### 2.2. Deposition of AZO Thin Films

Using RF magnetron sputtering, an AZO layer with a thickness of around 150 nm was deposited on the polymer substrates, which were thoroughly cleaned before being placed into the sputtering chamber. They underwent five minutes of ultrasonic cleaning in acetone, followed by ethanol, and, finally, deionized water. Without the addition of oxygen, heating the substrate, or post-annealing, the deposition procedure was carried out in an argon environment. The ceramic target used was AZO with 99.99% purity (98% ZnO and 2% Al_2_O_3_ by weight), having a diameter of 4 inches. It was positioned 20 cm away from the substrate within the chamber, which maintained a base pressure of 5.1 × 10^−6^ Pa. The deposition procedure used an argon flow rate of 50 sccm (standard cubic centimetre per minute at STP conditions), a constant RF power of 55 W, a power density of 0.7 W cm^−2^, a deposition pressure of 0.5 Pa, and a deposition rate of around 3.3 nm min^−1^. The target surface was thoroughly cleaned with argon plasma for five minutes as part of a pre-sputtering procedure.

### 2.3. Apparatus and Measurement

In order to assess the mechanical characteristics of both substrates, uniaxial stress was applied (Instron 4410). Utilizing a Jenway 6310 spectrophotometer, the optical transmittance of the films was determined across the visible spectrum between 400 and 800 nanometres. Furthermore, the resistivity of the film was assessed by using a four-point probe (Keithley 580 Micro-Ohmmeter). As detailed in [16], the residual stress imparted into the AZO layer during the deposition process was determined. Before subjecting the materials to testing, their surface microstructure and roughness were assessed using atomic force microscopy (AFM). The atomic force microscope (JPK Instruments, Cambridge, UK) was operated in contact mode. Si cantilevers with a nominal spring constant of 0.3 N m^−1^ were used for all imaging. By using X-ray diffraction (instrument and manufacturer), the structural features of the AZO/polymer substrate were also assessed.

**Figure 1:**
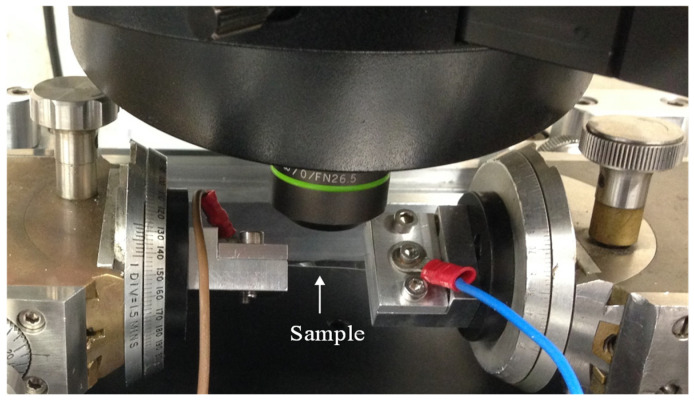
Custom-built twisting testing apparatus.

By using monotonic and fatigue twisting tests, the electromechanical flexibility of the AZO films coated to the polymer substrates was evaluated. A pre-existing apparatus [14], which is shown in Figure 1, was used for the monotonic twisting test. Copper wires were used to connect a FLUKE 45 multimeter to both grips so that the Lab View software could measure the electrical resistance in real time. Twisting started at 2° from the horizontal plane of the substrate and increased slowly until it reached about 62°. During a three-minute hold, images and changes in resistance were recorded at each 2° degrees. Each end of the sample had a grip that held it in place and turned it in opposite directions. A confocal laser scanning microscope (CLSM) was used to see how the cracks changed as the twisting angle increased. To study AZO film cracking, the crack density (CD) was measured at each step of the twisting angle. Using image analysis software ImageJ (NIH, USA) on optical microscope images, the CD was calculated. CD is equal to the reciprocal of the average distance between cracks in the AZO thin film.

Commercial rheometer equipment (ARES-G2) was used to perform twisting fatigue testing, using a technique similar to that reported by Mohammed et al. [14]. Two jigs were used to secure the sample; the bottom jig was coupled to a rotating driver, while the top jig was mounted to the stationary component of the rheometer. The bottom jig oscillated around the vertical axis throughout the test, causing the specimen to twist either clockwise or counterclockwise. Twisting sequences from 0° to 22.5°, back to 0°, then to −22.5°, and back to 0° made up each cycle. A multimeter was used to continually measure electrical resistance, and the Lab View software was used to record the results. Room temperature (RT) and twisting angles of ±22.5° were the experimental settings. After testing, the cracking morphology of the AZO films was examined using A JEOL JSM-7000F FE-SEM scanning electron microscopy (SEM). To improve electron conductivity, specimens were coated with a 5 nm thickness Au layer prior to analysis. The images were taken under an accelerating voltage of 15 kV and a working distance of 10 mm.

## 3. Results and Discussion

It was found that 150 nm AZO films deposited on PET and PEN substrate exhibited almost the same transparency, i.e., 80% in the visible wavelength spectrum, and a similar resistivity of 3.3 × 10^−2^ Ω cm. The transmittance and resistivity of AZO fabricated on polymer substrate were desirable values for the application of the thin film electrodes in flexible organic light emitting diodes and organic photovoltaics.

**Figure 2:**
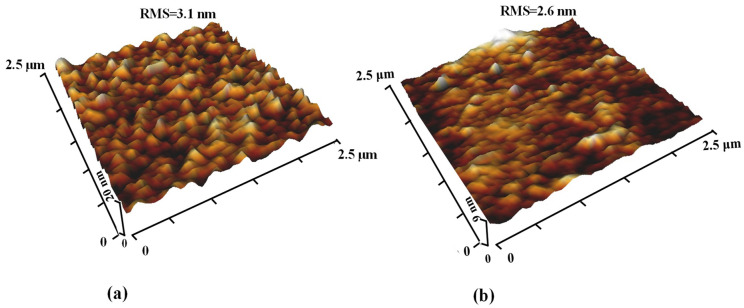
3D-AFM images of AZO films deposited on (**a**) PEN substrates and (**b**) PET substrates.

Figure 2 present AFM images that illustrate the surface morphology of a polymer substrate coated with AZO. The illustration in Figure 2 illustrates the existence of island-like formations dispersed throughout the AZO/PEN and AZO/PET film surfaces. In order to measure the surface roughness, root mean square (RMS) roughness values were collected from five separate locations on the film. It was determined that the mean RMS roughness value of the AZO-coated PET was approximately 2.6 nm. The measured value is considerably smaller when compared to that of AZO on PEN substrates, which is 3.1 nm. The observed discrepancy in surface roughness can be attributed to the intrinsically higher RMS roughness of the PEN substrate (1.6 nm) compared to the roughness of the PET substrate (1.1 nm). A minor variation in the crystallinity levels of the two substrates may account for the difference in surface roughness; thus, an increase in surface RMS values may be associated with the crystallization process. For applications involving flexible electronics, the uniformity of the electrode layer is emphasized as a critical factor, highlighting the significance of surface morphology in the performance and dependability of these materials.

**Figure 3:**
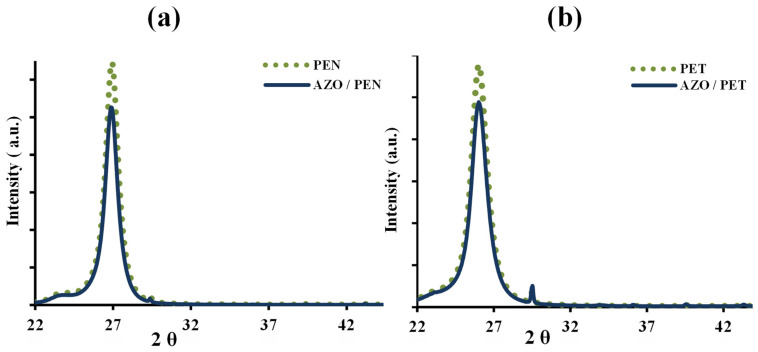
X-ray diffraction patterns of (**a**) AZO/PEN and PEN substrate, and (**b**) AZO/PET and PET substrate.

Figure 3a,b depict the XRD patterns of AZO-coated PEN and PET substrates, respectively. The XRD data for bare PET and PEN are included in Figure 3 for comparison. Sharp peaks in the patterns indicate the semi-crystalline nature of both polymer substrates. It is noted that compared to deposition on glass or silicon substrates, achieving crystallization of AZO thin films on soft substrates, such as PEN and PET, is challenging [17].

According to Leterrier et al. [16], the application of an ITO film onto a polymer substrate results in an increase in substrate temperature as a consequence of the energetic particle bombardment. This increase in temperature may cause compressive residual stresses in the AZO layer, as the polymer substrate undergoes subsequent cooling and contracts. An observed discrepancy in the compressive residual stresses between AZO/PEN (700 ± 187 MPa) and AZO/PET (1121 ± 215 MPa) was highlighted by the results. The observed variation may be ascribed to the distinct mechanical and thermal characteristics of the substrates composed of PEN and PET. In contrast to PET, PEN demonstrates enhanced bending rigidity and a higher glass transition temperature, which contribute to its increased resistance to dimensional changes at elevated temperatures [18]. As a consequence, the AZO/PEN sample experiences a significantly reduced residual stress. This may lead to lower thermal and mechanical mismatch between the AZO layer and the polymer substrate. Furthermore, the unique mechanical and thermal characteristics of PET and PEN are significantly influenced by their chemical structures [19].

**Figure 4:**
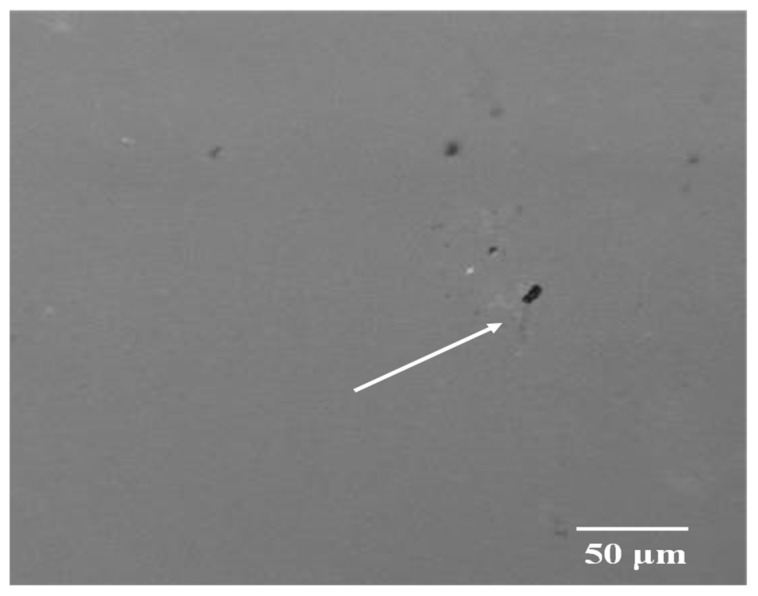
Displaying surface flaws and the initial cracks. The arrow indicates defect initiation sites.

An optical microscope was utilised to examine the surface of the polymer/coating composites throughout the deformation test. After careful inspection, it was noted that the as-deposited AZO coatings displayed defects on their surface, as illustrated in Figure 4.

**Figure 5:**
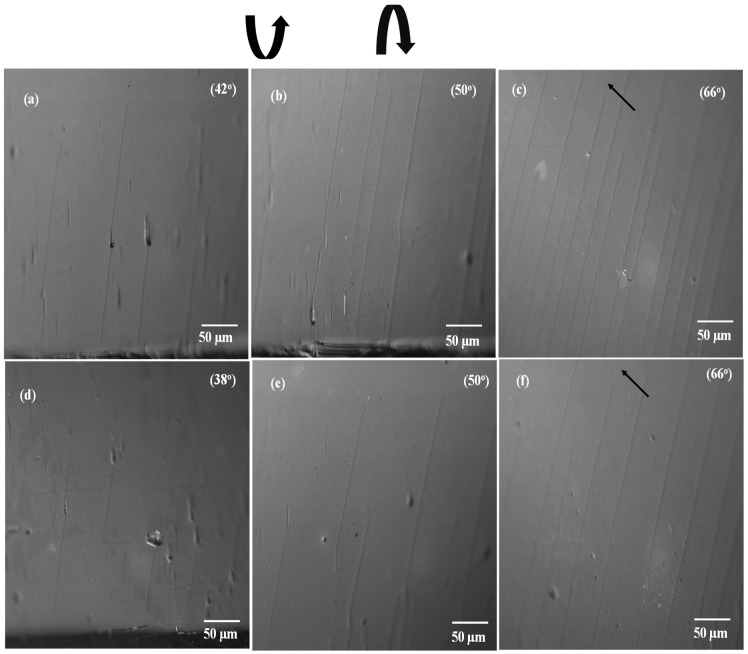
Crack patterns observed on AZO-coated PEN substrate (**a**–**c**) and PET substrate (**d**–**f**) with increasing twisting angles. Twisting arrows denote the direction of applied load, and the corresponding twisting values are indicated in the optical micrograph. Black arrows highlight areas where cracks have not fully propagated across the sample width.

These defects functioned as nucleation sites for the initiation of cracks. The CLSM micrographs presented in Figure 5a,b,d,f depict, at different angles, the initiation and progression of cracks in the AZO/PEN and AZO/PET samples, respectively.

Cracks were first observed at around 42° ± 2.1° and 38° ± 1.7° of twisting angle for AZO/PEN and AZO/PET, respectively. The small difference may be attributed to the lower mechanical mismatch between the AZO coating and the PEN [4,20]. Therefore, the AZO/PET sample cracks at a reduced twisting angle. The expansion and propagation of the initially formed channel fracture along the entire length of the sample are induced by augmenting the twisting angle that is applied. As illustrated in Figure 5f, it was noted that the channel crack does not traverse the complete breadth of the film. Rather, it terminates at a distinct distance from the sample width’s midpoint. This indicates that a maximal critical stress has been observed to occur in that specific region.

**Figure 6:**
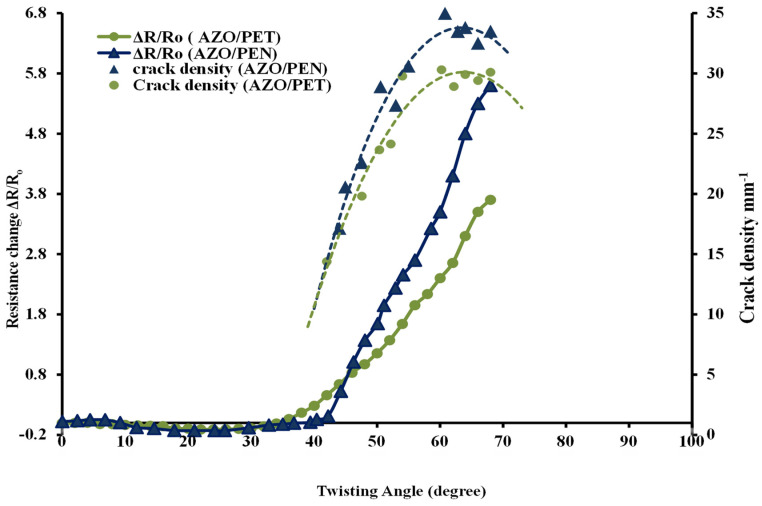
Crack density and change in resistance (ΔR/Ro) plotted against substrate twisting angle for AZO coatings on PEN and PET substrates.

Figure 6 depicts the evolution of fracture density and alterations in electrical resistance (ΔR/R_o_), where R_o_ denotes the electrical resistance value before the application of stress and R signifies the value subsequent to the application of twisting angle. Initially, when a twisting angle is applied, the normalized electrical resistance values are negative. This effect may be due to the decrease in the spacing between neighboring atoms or grains in the thin film. This reduction can lower physical obstacles to electron flow, thus improving electron mobility. The results are consistent with those documented by Potoczny [21] for ITO films on PET subjected to compressive stress. It indicates that the sample experienced compressive stress while undergoing the twisting tests.

As the twisting angle increases further, the density of cracks also increases correspondingly. In both scenarios, the electrical resistance increases, which is consistent with the results of prior research [14,22] concerning the deposition of thin ceramic coatings onto flexible substrates. As illustrated in Figure 5, this increase in resistance is due to the applied load-induced formation of cracks, which correlates with the film’s cohesive strength. The resistances of AZO/PEN and AZO/PET increase at twisting angles of approximately 43.6° and 39.4°, respectively, which are indicative of fracture initiation (see Figure 5). Furthermore, a higher saturated fracture density was noted in AZO/PEN samples relative to AZO/PET samples. This suggests that when AZO is applied to a highly rigid polymer substrate, it generates a greater number of cracks in an effort to relieve the applied stress. On the contrary, stress concentration can result in the establishment of debonding between a less rigid polymer substrate and AZO, whereby the stress is absorbed by the polymer substrate within the debonded region. Tsubone [23] has previously documented comparable results pertaining to the application of diamond-like carbon coatings onto polymer substrates.

**Figure 7:**
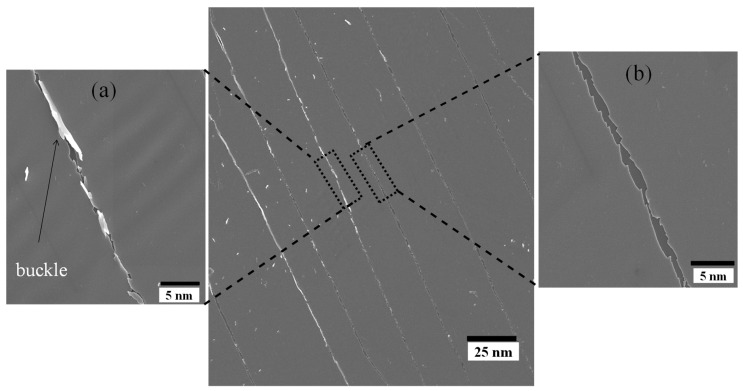
SEM micrograph with inset illustrating (**a**) overlapping and buckling delamination of AZO, and (**b**) surface cracks on the AZO film at a twisting angle of 68°.

One possible reason for the finite electrical resistance observed in AZO/polymer substrates, even under relatively high twisting angles, may be due to incomplete traversal of channel cracks across the sample width, as shown in Figure 5f, thereby allowing the sample to maintain its electrical conductivity. Moreover, the presence of overlapping film material at crack sites following crack formation (refer to Figure 7) contributes to the formation of conductive pathways between AZO films, leading to finite electrical resistance. This observation is consistent with a previous study by Jung et al. [14], which noted that during twisting, the overlap of cracked film facilitated the creation of a conductive pathway through fragmented film, resulting in delayed changes in electrical resistance.

Further insight into the failure mechanisms of the AZO thin film is provided by ex situ scanning electron microscopy. For example, as depicted in Figure 7a, the film experiences complete separation in certain areas due to the formation of wide cracks. However, in other areas, overlapping film and buckling delamination of the multilayer-stacked AZO thin film from the substrate are observed, as shown in Figure 7b. This observation is consistent with the alteration in electrical resistance data observed after reaching the crack saturation point.

The surface morphology of the twisted AZO film observed in this study (see Figure 7) closely resembles the coating damage resulting from tensile and compressive strain. These findings suggest that twisting deformation induces both tensile and compressive stress on the film simultaneously. A plausible explanation for the limited electrical resistance observed in AZO/polymer substrates, even when subjected to relatively high twisting angles, could be the incomplete passage of channel cracks along the width of the sample (illustrated in Figure 5c,f). This would enable the sample to retain its electrical conductivity. Furthermore, the formation of conductive pathways between AZO films is facilitated by the presence of overlapping film material at fracture sites subsequent to crack formation (see Figure 7). This ultimately results in finite electrical resistance. This observation aligns with the findings of a prior investigation conducted by Jung et al. [15], wherein they observed that the overlap of fractured film during twisting enabled the formation of a conductive pathway through fragmented film, leading to postponed alterations in electrical resistance.

**Figure 8:**
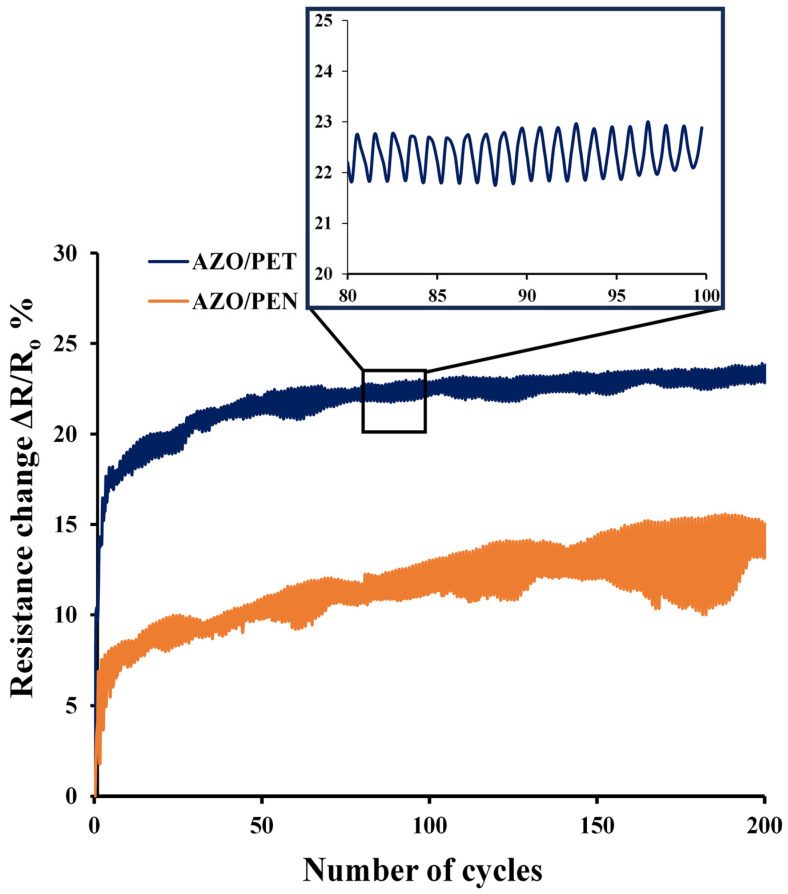
Resistance variation during the cycling twisting test as a function of the number of cycles.

Twisting fatigue tests were conducted for 200 cycles at a frequency of 12 s per cycle, with a consistent twisting angle of 22.5 degrees. Figure 8 illustrates that the resistance varied depending on the number of cycles for both AZO/PEN and AZO/PET samples. After the first few cycles, there was a significant rise in normalized electrical resistance, stabilizing at the 10th cycle for both samples. This may be attributed to the dimensional change in the polymer substrate [24,25]. Resistance changes eventually stabilized and then steadily increased with more twisting cycles applied.

After 200 cycles, the resistance of the AZO/PET sample increased by around 23%, which was half the increase recorded in the AZO/PEN sample (roughly 13% at the same twisting angle). The smaller rise in normalized electrical resistance during cycle tests of AZO/PET samples may be due to the decreased mechanical mismatch between an AZO film and a PEN substrate, resulting from the higher elastic modulus of PEN in comparison to PET.

No SEM images of the AZO/PEN and AZO/PET samples after the cyclic loading test under twisting were available since no obvious cracks were found on the sample surfaces. Submicron cracks may exist in the AZO film, as shown by the steady increase in resistance observed during the test with a growing number of cycles. The absence of visible cracks in the coating may result from the strain recovery that occurs after unloading the sample, causing microcracks to close [24].

## 4. Conclusions

Our study investigates the integration of amorphous AZO films, each 150 nm thick, deposited onto PEN and PET substrates using RF magnetron sputtering at room temperature. The films demonstrate low electrical resistance of approximately 3.3 × 10^−2^ Ω cm and high transparency of around 80% in the visible range. We evaluated the flexibility of AZO/PEN and AZO/PET samples under both monotonic and cyclic twisting conditions.

Our reliability studies reveal that the electrical resistance of AZO on PEN remains stable up to twisting angles of approximately 42°, while for AZO on PET substrates, it remains unchanged up to 38°. AZO films on PEN substrates exhibit significant saturation crack density due to mechanical mismatches between the coating and substrate. SEM investigations show that failure mechanisms include cracking, buckling, and delamination, indicating induced tensile and compressive stresses from twisting motions.

In twisting fatigue experiments, resistance analysis demonstrates an increase with the number of cycles, attributed to dimensional changes until reaching a stable width, followed by gradual linear growth possibly due to AZO cracking. These findings provide insights into the failure mechanisms of AZO thin films on polyesters under various twisting deformations, offering guidance for flexible optoelectronic device design. 

## Data Availability

The original contributions presented in the study are included in the article, further inquiries can be directed to the corresponding author.
